# Late-Onset Huntington’s Disease: A Case Report and Literature Review

**DOI:** 10.7759/cureus.102298

**Published:** 2026-01-26

**Authors:** Carlos Gonçalves, Ana Sofia Ferreira, André Calheiros, Rafael Lopes Freitas, Goncalo Cacao

**Affiliations:** 1 Internal Medicine, Unidade Local de Saúde do Alto Minho (ULSAM) Hospital Conde de Bertiandos, Ponte de Lima, PRT; 2 Intensive Care Medicine, Unidade Local de Saúde de Matosinhos, Porto, PRT; 3 Neurology, Unidade Local de Saúde do Alto Minho (ULSAM) Hospital de Santa Luzia, Viana do Castelo, PRT

**Keywords:** chorea, cognitive decline, huntington’s disease (hd), late-onset huntington’s disease, neurodegenerative disease

## Abstract

Huntington’s disease (HD) is a rare autosomal dominant neurodegenerative disorder caused by expansion of the cytosine-adenine-guanine (CAG) trinucleotide repeat in the huntingtin (HTT) gene. Although the disease typically presents in mid-adulthood, symptom onset after the age of 60, defined as late-onset Huntington’s disease (LoHD), remains uncommon and may pose diagnostic challenges. We report the case of an 80-year-old man admitted for evaluation of progressive unintentional weight loss, whose clinical assessment revealed generalized chorea and progressive cognitive decline. Genetic testing identified an expanded CAG allele with 39 repeats, confirming the diagnosis of LoHD. Neuroimaging revealed ischemic leukoencephalopathy consistent with cerebral small vessel disease (CSVD), contributing to diagnostic complexity. This case highlights the importance of considering Huntington’s disease in the differential diagnosis of late-onset chorea and cognitive impairment, even in the absence of a known family history.

## Introduction

Huntington’s disease (HD) is a rare autosomal dominant neurodegenerative disorder characterized by abnormal expansion of the cytosine-adenine-guanine (CAG) trinucleotide repeat in the huntingtin gene (HTT), leading to the production of a mutant huntingtin protein with toxic properties [1]. This results in synaptic dysfunction, transcriptional dysregulation, oxidative stress, and neuronal loss, predominantly affecting the striatum and giving rise to the classical clinical triad of motor, cognitive, and psychiatric manifestations [1]. The most common motor presentation is chorea, although rigidity, dystonia, and oculomotor abnormalities may also be observed [1].

The number of CAG repeats is the principal determinant of disease penetrance and age at onset. Alleles with ≥40 repeats are associated with complete penetrance and earlier disease onset, whereas alleles with 36-39 repeats demonstrate reduced penetrance and greater phenotypic variability [1]. Nevertheless, modifying factors, including deoxyribonucleic acid (DNA) repair genes such as MutS homolog 3 (MSH3) and Fanconi anemia-associated nuclease 1 (FAN1), significantly influence age at onset and disease progression, underscoring the complexity of the pathogenic mechanisms involved [2].

Late-onset Huntington’s disease (LoHD), defined as symptom onset after 60 years of age, has been reported to account for approximately 4.4%-11.5% of HD cases [3]. In this subgroup, the clinical phenotype is often more heterogeneous and frequently dominated by cognitive impairment or behavioral changes, while chorea may be less prominent. This contributes to diagnostic difficulty, particularly in differentiating LoHD from more prevalent neurodegenerative dementias and parkinsonian syndromes in older adults [2,4]. In older adults, clinical recognition may be further delayed by overlap with age-related neurodegenerative and vascular conditions (including cerebral small vessel disease (CSVD)), which may confound the interpretation of cognitive decline and functional deterioration [3,4]. Recent retrospective cohort data also highlight substantial diagnostic delay in LoHD, reinforcing the challenges of recognition in older adults [5]. Additionally, up to one-third of patients with LoHD do not report a clear family history, commonly due to reduced-penetrance alleles or unrecognized familial disease [4].

In advanced stages of HD, irrespective of age at onset, there is a transition toward a predominantly hypokinetic phenotype, characterized by rigidity, bradykinesia, and significant functional decline [1]. Cognitive deterioration progresses to a subcortical dementia with marked executive dysfunction and apathy. Nutritional complications, respiratory infections, and dysphagia become major contributors to morbidity and mortality [1]. In patients with LoHD, these features often overlap with age-related comorbidities, making a careful and multidisciplinary diagnostic approach essential [4].

## Case presentation

An 80-year-old man with a history of arterial hypertension and no known family history of neurological or psychiatric disease was admitted for investigation of constitutional symptoms, including progressive unintentional weight loss. According to his family, involuntary movements had been present for approximately four years, initially mild and of small amplitude in the left foot, gradually progressing to involve all four limbs, the trunk, and the face, becoming increasingly pronounced. Over time, the patient developed progressive loss of autonomy in ambulation, personal hygiene, and feeding, concomitant with continued weight loss. In the months preceding admission, he also exhibited episodes of disorientation, behavioral changes, and fluctuations in attentional state.

**Figure 1:**
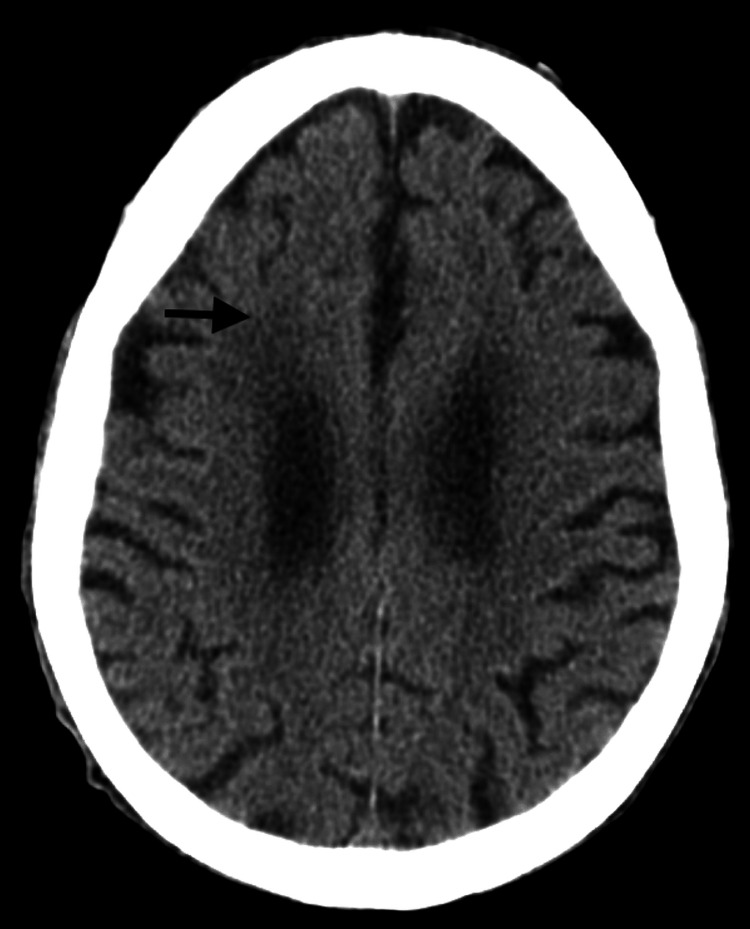
Non-contrast cranial computed tomography demonstrating ischemic leukoencephalopathy (arrow)

On physical examination, the patient was markedly underweight, with generalized chorea and multidomain cognitive decline. Laboratory investigations (Table 1) revealed acute kidney injury of prerenal etiology, interpreted in the context of dehydration in a patient with reduced oral fluid and nutritional intake, without associated electrolyte disturbances. Cranial computed tomography (Figure 1) demonstrated ischemic leukoencephalopathy. No alternative cause for the weight loss was identified, and it was attributed to pronounced chorea associated with inadequate nutritional intake.

**Table 1:** Laboratory test findings on admission and at discharge from the hospital

Laboratory test	Observed value		Normal range
	On admission	At discharge	
Hemoglobin (g/dL)	13.2	13.4	13.2-17.2
Creatinine (mg/dL)	3.35	0.61	0.60-1.30
Urea (mg/dL)	338	27	17-43
Sodium (mmol/L)	138	137	136-145
Potassium (mmol/dL)	3.9	5	3.5-5.1
C-reactive protein (mg/dL)	<0.51	<0.51	<0.51

Given the suspicion of Huntington’s disease, a neurology consultation was requested, and a therapeutic trial with haloperidol was initiated, resulting in partial improvement of the involuntary movements. Genetic testing revealed one expanded CAG allele with 39 repeats and one normal allele with 11 repeats, confirming the diagnosis of Huntington’s disease. The patient remains under follow-up with symptomatic control achieved through neuroleptic therapy and restoration of adequate nutritional intake, and the family has been referred for genetic counseling.

## Discussion

Late-onset Huntington’s disease (LoHD), typically defined as symptom onset after 60 years of age, remains an uncommon phenotype but is increasingly recognized. In older adults, diagnostic suspicion may be low, and clinical presentation can overlap with age-related neurological disorders, contributing to diagnostic delay. In a large retrospective cohort from Mexico, LoHD represented 7% of genetically confirmed Huntington’s disease cases, with a mean diagnostic delay of 6.2 years, emphasizing how frequently recognition is postponed in this subgroup [5]. In our patient, symptom onset in the eighth decade, together with prominent weight loss and progressive cognitive decline, contributed to diagnostic uncertainty and delayed identification.

Genotype-phenotype correlations are particularly relevant in LoHD. Expansions in the reduced penetrance range (36-39 CAG repeats) are associated with increased variability in clinical expression and later onset compared with fully penetrant expansions (≥40 repeats) [1,2]. The identification of 39 CAG repeats in this case aligns with the observation that LoHD often involves lower-range pathological expansions and may follow a more insidious disease evolution [3,4]. However, CAG repeat length alone does not fully account for age at onset, and multiple genetic and environmental modifiers influence disease penetrance and clinical course, including DNA repair pathways [2].

From a clinical standpoint, Huntington’s disease is characterized by progressive motor, cognitive, and psychiatric manifestations [1,6]. In older adults, cognitive decline and functional deterioration may dominate the clinical phenotype, while chorea may be initially subtle and attributed to other causes, further complicating recognition [4,5]. Psychiatric and behavioral symptoms also represent a major component of Huntington’s disease and may occur early or be misinterpreted as primary psychiatric illness, particularly in late-life presentations [7]. In addition, the absence of an apparent family history should not exclude Huntington’s disease. In LoHD cohorts, a substantial proportion of patients have unclear transmission patterns or no known affected relatives, likely due to reduced penetrance alleles, incomplete family history, small family structures, or unrecognized disease in relatives [4,5]. This was similarly observed in our case.

Neuroimaging interpretation in LoHD requires particular caution. While Huntington’s disease is classically associated with striatal involvement, older patients frequently demonstrate coexisting cerebrovascular pathology. In our patient, cranial computed tomography revealed ischemic leukoencephalopathy consistent with cerebral small vessel disease (CSVD). MRI was not available in this case to better assess striatal (caudate) atrophy. CSVD is common in advanced age and is independently associated with cognitive impairment, gait disturbance, and loss of functional autonomy. Therefore, CSVD likely contributed to the severity of cognitive decline and functional deterioration in this case and was an important confounder during the diagnostic process. Nevertheless, the progressive generalized chorea, cognitive decline, functional worsening, and molecular confirmation of HTT CAG expansion strongly support Huntington’s disease as the primary unifying diagnosis.

Accordingly, the differential diagnosis of late-onset chorea with cognitive decline should be broad. In elderly patients, clinicians should consider neurodegenerative conditions (e.g., dementia with Lewy bodies, frontotemporal dementia, and corticobasal syndrome), medication-induced hyperkinesias, autoimmune/paraneoplastic syndromes, metabolic causes, and structural lesions. In this clinical context, the frequent coexistence of age-related disorders such as CSVD reinforces the importance of systematic differential diagnosis and recognition that vascular pathology may coexist and amplify disability without fully explaining progressive chorea and a neurodegenerative trajectory [5,8].

Prognostically, LoHD should not be regarded as necessarily benign. In a multicenter South Korean study evaluating survival in LoHD, clinical and genetic variables were associated with survival outcome, and survival after onset remained limited, supporting the substantial morbidity burden in this population [8]. In our case, marked weight loss likely reflected a combination of hyperkinetic movements, reduced nutritional intake, and frailty, highlighting the importance of early nutritional evaluation and supportive interventions.

Although disease-modifying therapies are not yet available, the diagnosis has meaningful clinical implications. Definitive diagnosis requires molecular confirmation of HTT CAG repeat expansion [1,2]. Early genetic confirmation allows timely initiation of symptomatic treatment (e.g., neuroleptics for chorea), multidisciplinary supportive care, nutritional and swallowing assessment when appropriate, rehabilitation strategies to reduce falls, and referral for genetic counseling [1,7]. A limitation of this report is the lack of publication-ready MRI images to better illustrate caudate nucleus involvement and characterize concomitant cerebral small vessel disease.

Finally, emerging evidence suggests that Huntington’s disease may include a neurodevelopmental component that contributes to circuit vulnerability later in life, further underscoring the complex biology underlying clinical expression across the lifespan [9,10].

## Conclusions

Late-onset Huntington’s disease is an uncommon but clinically important cause of progressive chorea and cognitive decline in older adults, even in the absence of a known family history. Given the frequent coexistence of age-related conditions such as cerebral small vessel disease, which may contribute to cognitive impairment and functional deterioration, careful differential diagnosis is essential. Definitive diagnosis requires molecular confirmation of HTT CAG repeat expansion, enabling appropriate symptomatic management, nutritional support, multidisciplinary care, and genetic counseling.
